# Trust and ambivalence in midwives' views towards women developing pelvic pain during pregnancy: a qualitative study

**DOI:** 10.1186/1471-2458-10-600

**Published:** 2010-10-12

**Authors:** Ingrid Mogren, Anna Winkvist, Lars Dahlgren

**Affiliations:** 1 Department of Clinical Science, Obstetrics and Gynecology, Umeå University, Umeå, Sweden; 2 Department of Metabolism and Cardiovascular Research/Clinical Nutrition, Sahlgrenska Academy, Gothenburg University, Gothenburg, Sweden; 3 Department of Sociology, Umeå University, Umeå, Sweden

## Abstract

**Background:**

The Swedish midwife plays a significant role in the antenatal care (ANC) system, and a majority of pregnant women are satisfied with their ANC. Pelvic pain during pregnancy (PP) is prevalent. The study investigated the views, perceptions and attitudes of midwives currently working in ANC regarding PP during pregnancy.

**Methods:**

The informants were ten midwives between the ages of 35 to 64 years, with a combined experience of 250 years of midwifery. In-depth interviews (n = 4) and one focus group discussion (n = 6) were conducted. The data were interpreted using a qualitative content analysis design.

**Results:**

PP was considered a common, clinical problem that had most likely increased in prevalence in recent decades and could feature prominently in a woman's experience of pregnancy. The informants had developed a strategy for supporting pregnant women affected by PP. The pregnant woman's fear of not being believed concerning her symptoms and the risk of being regarded as a malingerer were acknowledged. Mistrust between a midwife and a woman might occur when the patient's symptoms were vague and ill defined. PP was not considered as something that complicated delivery, and women experiencing it were advised to await 'the natural course of the pregnancy'.

**Conclusions:**

PP was considered a common, clinical problem and the informants had developed a strategy for supporting pregnant women affected by PP. However, the woman's fear of not being believed concerning her symptoms of PP was acknowledged and mistrust might occur between a midwife and a woman if vague symptoms were reported.

## Background

Antenatal care (ANC) in Sweden is free of charge, easily available, and used by almost all pregnant women [1]. The Swedish routine programme for ANC entails several scheduled visits for detecting maternal and fetal complications and providing psychosocial support and health education; however, the scientific evidence supporting the timing and contents of the routine visits has been questioned [2,3]. The Swedish midwife plays a significant role in the ANC system, working within the primary health care system but in close collaboration with obstetricians in hospital-based care [4]. In general, Swedish women have high expectations of ANC in relation to prevention of fetal morbidity [5]. A vast majority of Swedish women (87.6%) are satisfied with the overall ANC received, but less so with its emotional (76.9%) than with its medical (82.3%) aspects [6]. From a woman's perspectives important characteristics of the midwife are being supportive, friendly, attentive, respectful and nonjudgmental [7]. Trust is an important concept for the caring disciplines, such as for example nursing and medicine. It is built over time between the caregiver and the patient [8] and has been identified as an important element in the nurse-patient relationship [9]. The centrality and quality of relationship have been increasingly addressed by research focusing on maternity care and the midwife-woman relationship [10-12].

The midwives' and expectant parents' ways of relating to each other have been investigated qualitatively; the midwives appear to direct the consultations, generally applying a basic pattern in the interaction, though occasionally applying other patterns as well [13,14].

Most studies report that over half of the pregnant population suffers from pelvic pain (PP) and/or back pain during pregnancy [15-18], which indicates that the problem has to be dealt with during ANC consultations. Despite its high prevalence, the aetiology of the condition is still unknown [19], and furthermore, there are no curative measures to offer to pregnant women. There is no consensus as to the specific criteria of the condition, although attempts have been made in that direction [19].

This study focuses on one of the categories of health professionals who encounters pregnant women suffering from pelvic pain, namely midwives. Above all, we will call attention to the midwives' understanding of this interaction and how they deal with the condition and those suffering from it. Because the existence of pelvic pain has been questioned, both by laypeople and in medical discourse, discussion concerning it may be complicated and sensitive. The patients expect that health care professionals, in this case, midwives, will provide a clear diagnosis, appropriate information and suggestions for relevant treatments. The Swedish socio-anthropologist, Lisbeth Sachs [20], has analysed the phenomenon in terms of trust, and stated that trust is a crucial property in encounters within medical care. Referring to researchers such as Harrington and Brody [21,22], Sachs emphasises the importance of diagnosis, but states that the diagnosis should be done by a trusted person, preferably a professional and at the same time a trusted fellow being. Where there is trust the patient tends to feel comfortable. There are thus two sides to a patient's trust in a doctor or midwife: these professionals may be trusted because of their perceived medical competence, but also because of their empathy and human kindness [20]. However, the trust of the caregiver towards the patient has not been examined as extensively in the literature.

This study investigated the views, perceptions and attitudes of midwives currently working in ANC, regarding pelvic pain during pregnancy. To the best of our knowledge, this is the first study addressing these research questions.

The study was approved by the Ethics Committee of Umeå University (Case no. 01-249).

## Methods

Initially, four midwives currently working in ANC within one catchment area in northern Sweden were purposively and consecutively asked to participate in the study, and were verbally informed of the aims and consequences of participation. The informants were interviewed at a location of their choice. All informants were females aged between 35 and 58 years and were approached based on age and work experience as midwives. Their individual professional experience of midwifery ranged from 6 to 35 years, and their collective experience totalled 94 years. The informants had experience of working in both ANC and in delivery wards. The individual in-depth interviews were collected from 2002 to 2004, and the interviews were conducted by two of the authors, three informants being interviewed by Lars Dahlgren (LD) and one by Ingrid Mogren (IM) and they were all interviewed on one occasion. The length of the in-depth interviews varied between approximately 60 to 90 minutes. In order to increase the transferability, a second data collection (focus group discussion, FGD) was conducted in January 2007 in another catchment area of northern Sweden. Six female midwives were recruited and their ages ranged between 45 and 64 years. Their collective professional experience as midwives totalled 156 years. They were all working within ANC at the time. None of the subjects participated at both data collections. The length of the FGD was 105 minutes. The interviewers were LD and IM.

A thematic interview guide was used with the in-depth interviews as well as at with the FGD. This guide included questions and themes such as 'how do you experience the condition pelvic pain during pregnancy?', 'what is your view on the maternal significance of developing pelvic pain during pregnancy', 'management of women developing pelvic pain during pregnancy - health seeking behaviour and obstacles to care' among others.

Data collection continued until we observed that our data had reached the level of redundancy [23], meaning that our categories and theoretical constructs were saturated. The interviews and the FGD were transcribed verbatim. The principal investigator, IM (female), is an obstetrician with a special clinical interest in pelvic pain during and after pregnancy. The co-investigators, AW (female) and LD (male), are both active in the research field of public health. AW is a nutritionist with a research focus on reproductive health, while LD is a medical sociologist with a research focus on the sociology of emotions.

Data interpretation used qualitative content analysis [24] as developed in nursing research [25]. This implies attempts to interpret both manifest and latent contents of the data. The first step implies the coding of the data, i.e., the construction of tools aimed at labelling the interview texts so as to allow them to be considered in new ways [25], thus generating a categorisation of the views, attitudes and perceptions of the informants. The second step was to aggregate the codes into exhaustive categories intended to describe the issues under study. Finally, we identified themes that encompassed the meanings of the text and merged the categories in a more theoretical way. With help of these categories and themes, we attempted to construct a web or pattern with which to improve our understanding of interaction between midwives and pregnant women in the context of PP during pregnancy.

## Results

Seven different themes emerged during analysis, as follows: i) To trust or not to trust - is pelvic pain normal during pregnancy?; ii) Has there been an increase in pelvic pain during pregnancy?; iii) Probable risk factors for pelvic pain during pregnancy; iv) 'The joints are cracking, snapping, and clicking'; v) The maternal pelvic pain scenario vi) Being supportive enough; and vii) What women may expect in subsequent pregnancies. These themes are described and discussed below.

### To trust or not to trust - is pelvic pain during pregnancy normal?

The overall experience of the midwives was that pelvic pain during pregnancy (PP) was a common, clinical problem that may constitute a health problem for the pregnant woman, thus playing an important role in her experience of pregnancy. One informant had formerly long seriously doubted whether PP really existed and could be labelled a medical condition. The informants had been exposed to various opinions from other health care workers regarding this issue; however, most of these workers had eventually come to agree on the existence of PP.

Some level of back pain or pelvic pain during pregnancy was regarded as normal and physiological in pregnancy, and doubts were voiced as to whether pregnant women were being falsely diagnosed with PP by themselves or others. The diagnostics mainly involved the assessment of typical symptoms, but tests could also be performed to confirm the diagnosis, although they were seldom used by the midwives.

Women who contracted PP shared no obvious prominent characteristics. If PP started late in pregnancy, it was often experienced as a minor problem and something expected by her. An extremely early onset of symptoms, however, might be interpreted as associated with false symptoms, though it could also indicate the possibility of the later development of severe PP.

You want to trust everyone. However, sometimes the symptoms are so diffuse or not really consistent with ordinary pelvic pain symptoms, that although she [the patient] claims it [i.e., pelvic pain], you may become doubtful.

Pelvic pain was thus recognised as a condition that could develop and become severely debilitating during pregnancy, although such extreme cases were fairly rare. One informant reasoned that pelvic pain symptoms might represent a way of communicating with the environment, and that the pain symptoms reflected the patient's life situation, general life stress, or a distorted view of the patient's own body image.

### Has there been an increase of pelvic pain during pregnancy?

The overall impression was that PP has become more prevalent among pregnant women, and that the condition was better known by the public than before. The increase in prevalence was perceived as having accelerated over perhaps the past ten to fifteen years, and the condition was now assessed as affecting approximately half the pregnant population.

I don't remember that I met pregnant women with PP at the beginning of my career, though I had heard of the condition.

Today I encounter it almost every day.

They were so few when I started as a midwife 35 years ago.

Obviously, it has increased over the years.

It was suggested that it now may be more acceptable for pregnant women to say that they are experiencing back pain and PP. It was also hypothesised that the previous 'low prevalence' of PP among pregnant women could be attributed to a previous lack of a label for the condition, implying that the medical profession had simply not discovered an existing condition.

### Probable risk factors for pelvic pain during pregnancy

Factors such as physical inactivity, bad posture, increased weight, incorrect body loading, physically demanding and monotonous work, back pain before pregnancy, and lower muscular strength were cited as probable risk factors for the development of PP. There were some contradictive views regarding physical activity in the general, female population. On one hand, modern-day women were described generally less active; on the other hand, other modern women were described as more physically active in various athletic activities associated with excessive stretching of the ligaments, which would result in an unstable pelvis.

Even those who are in very good physical condition may develop PP.

The pregnancy-induced relaxation of the pelvis was regarded as a normal temporary state and adaptation during pregnancy. Women contracting pelvic pain were also regarded as, to some extent, more ambitious and occupied with many different tasks in their lives; thus, an increased pace of life was assumed to be associated with PP.

### 'The joints are cracking, snapping, and clicking.'

The symptoms of pelvic pain were considered as fairly simple to interpret because of their consistency from patient to patient.

They tell very clearly how they perceive their pain symptoms. I think it's obvious.

The common case was that of a nulliparous woman with the onset of symptoms fairly late in pregnancy. Parous women generally had an earlier onset of pain, and the symptoms could more easily develop to a more severe level. Besides the pain symptoms, the condition could also involve the joints 'cracking, snapping, and clicking' and pronounced pelvic instability. Women experiencing more severe symptoms also often suffered from associated tiredness.

### The maternal pelvic pain scenario

The informants agreed that a common feature of women with PP was their fear of not being believed and of being regarded as malingerers. This fear of being disbelieved applied to the health care system, and the patients' professional contexts and sometimes even family. The intensity of the pain was recognised as varying from day to day, a feature considered as likely to impede understanding of the women by their environment.

They have better and worse days and if she one day may let the crutches go, yes, then the others ask how come? That is one of the difficulties with the condition that it is not permanently at the same level all the time.

Pelvic pain could also negatively influence the mood of the pregnant women and make them downhearted. The joy of pregnancy could be decreased, and the women might express feelings of individual failure because of PP. The women could also have feelings of guilt towards their babies because they could not enjoy being pregnant as they perceived they should. However, the pain did not prevent the growth of love for the baby. Some women - because of the high level of pain - reached a limit where they could not bear being pregnant any longer. For most women, however, PP was perceived as something transient, a condition only occurring during pregnancy.

Women with moderate to severe PP were considered to be unable to manage parts of their household work, and the same held true for their professional work. In severe cases, PP was considered as seriously debilitating. PP was then associated with restrictions in movement, ordinary activity, and work tasks and the women experienced tension between what they wanted to do and were able to do in their ordinary lives. The women could be encouraged to find alternate sexual activities because of aggravated pelvic pain provoked by intercourse.

When developing PP, the women initially often reacted with, for example, incomprehension as to why *they *had contracted the condition. They had difficulties accepting that their performance was not at the same level as before in their professional and private lives.

Some women have difficulties accepting that they cannot continue their professional activities as previously.

However, with time most women accepted the new situation and adapted to the new circumstances.

### Being supportive enough

The informants found that most pregnant women already had some knowledge of PP, and some informants provided no information regarding PP unless the pregnant woman had symptoms. Although the midwives acknowledged the need for information and discussion of PP, the time limits of regular visits restricted what could be dealt with in relation to PP, such as for example sexual life during pregnancy. In some cases there was uncertainty as to whether the pregnant woman really had contracted PP, even though the classical symptoms were reported; in such cases mistrust had clearly occurred. The midwives' own knowledge on PP was mainly self-acquired through their own experiences.

If a pregnant woman developed PP, the midwife gave counselling that, in the initial stage, mostly consisted of information regarding the condition and advice on how to deal with it. The content of the counselling was adjusted to the individual. The advice mainly concentrated on limiting the further progress of the condition, and included information on symptomatic regimens such as use of the pelvic belt, transcutaneous nerve stimulation (TENS), acupuncture, physiotherapy, and various pharmacological pain-relief methods. Acupuncture was considered as the best method to enable the women to achieve a fairly 'normal' life despite their PP.

They had less pain and some became almost painless. However, it is important that the women take care of themselves even during treatment [i.e., acupuncture]. They should not take that long walk just because they are painless, because they will pay for it some other day.

There are many more [acupuncture] treatments to go. However, my patients do not complain, since they do it in order to feel a little better some day.

It [acupuncture] meant she could have a fairly normal life without crutches or a wheelchair.

Other methods were considered less effective. The women were encouraged to engage in physical activities, such as in-water exercise and swimming. It was also recognised that the condition could not be cured by physical activity.

The importance of support and understanding of the woman from her social environment and from the ANC system was underlined. Thus, the informants believed that the pregnant women's partners should also receive appropriate information regarding the condition. Referrals to psychologists or welfare officers were seldom needed. The informants made adjustments of the predetermined consultation schedule depending on the needs of the women, for example, those connected with PP. One midwife said that simply talking about pelvic pain would not improve the condition. When PP progressed, the midwife could help with practical arrangements such as pelvic belts or crutches. If mild PP developed into moderate or severe symptoms, the women affected could be referred to a physiotherapist or physician for assessment and further treatment.

The informants reported that most pregnant women were granted sick leave at some point during their pregnancies (irrespective of cause of sick leave), and that a small proportion of women were probably granted sick leave on false terms.

Yes, you may get critical and wonder whether they really are eligible for sick leave. However, when I consider my own patients I don't think that there are many of them who fake [symptoms].

I think that they need their sick leave because you cannot function in your ordinary life if you have moderate or severe PP.

Half-time sick leave was regarded as beneficial for women with pelvic pain, as it allowed them to work less hard and to rest somewhat, while maintaining continuity in relation to their social context. Some pregnant women with severe PP were described as in despair, and it was sometimes evident that the woman did not believe that the midwife was inclined to help. Midwives could come to feel helpless in the face of pregnant women with severe PP problems.

The informants believed that PP did not contraindicate vaginal delivery, and they felt it was important to support and convince the pregnant woman that the delivery would most likely be successful despite the pelvic pain.

The way I have understood it is that it is not the delivery which is the problem; rather, it is the pregnancy that places the major strain on the pelvis.

As some women had severe symptoms, the issue of induction of labour, to shorten the pregnancy, was discussed. The midwives generally felt that PP *per se *was not associated with specific complications during pregnancy and delivery; however, if induced labour was chosen because of severe PP, the women would face the same risks faced by other women undergoing induced delivery. Furthermore, use of epidural anaesthesia was considered as potentially negative for PP, since the pain symptoms were considered as a protection against inappropriate positions that could aggravate PP.

I try to encourage them to await spontaneous labour and let nature take its course.

At the regular check up after the delivery (at approximately eight weeks post-partum) most women who had experienced PP were in remission. PP was seldom discussed thoroughly at this final visit. However, in some cases the women announced that they would not get pregnant again because of their experiences of PP during pregnancy.

### What women may expect in subsequent pregnancies

It was assumed that PP would worsen with every subsequent pregnancy. Experiencing PP might prompt a woman to decide to avoid future pregnancy. However, some women had an urgent longing for more children, despite their very negative experiences of pelvic pain during previous pregnancies, and therefore decided to endure another pregnancy. In severe cases, in which the patient had been restricted to a wheelchair, the patient was less likely to plan for a subsequent pregnancy, and that was considered as an active choice by the woman. However, the informants did not in general consider PP during pregnancy as a contraindication to subsequent pregnancy.

## Discussion

Midwives considered PP during pregnancy to be prevalent, with symptoms varying in severity, although they usually worsened during the course of the pregnancy. They also perceived that the condition was more prevalent today than before, though the cause of this development was unclear.

In general, Swedish midwives find their work to be satisfying and significant, as it includes the possibility of variation and offers profound contact and the possibility of ongoing relations with prospective parents [12]. In turn, most Swedish pregnant women are satisfied with their ANC [6]. Maintaining health and treating pregnancy as a natural condition are the declared policy aims of Swedish midwives [26]. It has been suggested that two different discourses exist in modern maternity care, namely, 'the medical science of birth' and the 'traditionally based knowledge' (including a natural perspective), and that midwifery is practised at the intersection of these two discourses [27,28]. Three basic patterns have been recognised in how the Swedish midwife and the pregnant woman/couple relate to each other in maternity care consultations [14]. These basic patterns include the roles of 'the respectful gardener and her developing plants', 'the propagandist teacher and her ignorant pupils', and 'the controlling inspector and the representatives of the population at her disposal' [14]. In the same study, two other patterns were recognised in addition to these basic ones, namely, 'the mediating consultant and the discreet seekers of guidance' and 'personal women friends'. We did not investigate this issue, although features of the described relationship patterns could be recognised among our subjects. Midwives are found to be committed at both the professional and personal levels, and that advocating characterises the nursing profession [12]. The need for advocacy was especially cited by our informants at the second data collection. The advocacy was directed mainly towards representatives of the local Social Insurance Board. A contrasting finding in relation to the initial data collection was that the general opinion at the FGD was that pregnant women only exceptionally were granted sick leave because of pelvic pain during pregnancy. This difference might have been attributed to policy alterations in sick leave management between the first and the second time period or due to different policies at the local Social Insurance Boards.

The essence of midwifery when caring for women at high obstetric risk or with a manifested complication has been defined as 'a struggle for the natural process' [28]. Since PP *per se *was not considered as likely to complicate delivery, pregnant women were supported in awaiting the 'natural course of the pregnancy' and the situation was commonly regarded as not dominated by medical perspectives that aimed to intervene to end the pregnancy.

Mutual mistrust, in relation to PP, was mentioned as a problem that could arise between the midwife, and health care in general, and the pregnant woman. On one hand, the midwives were aware of the despair and distrust that could be experienced by the pregnant woman during encounters with ANC and the health care system. On the other hand, the midwives could themselves be hesitant when the described symptoms deviated from the ordinary clinical picture of PP. This situation might induce imbalance within the role of the midwife as a provider for the medical and psychological needs of pregnant women, and could also impair the relationship between midwife and patient. However, mistrust was not considered to be a major problem by the informants. The centrality of relationships within maternity care and nurse-patient relationships has recently been discussed [9,11] and central concepts in the midwife-woman relationship have been suggested [10]. One of these central concepts is 'trust-mediation of trust', which implies trust of oneself i.e. the pregnant woman, and mediation of trust, which means that midwives promote a trustful relationship [10].

Further, the informants developed a strategy of how to support pregnant women affected by PP; however, the different treatments and measures available could be perceived as insufficient. Since there are no curative measures that may be undertaken during pregnancy, symptomatic treatment is the only option and both counterparts were aware of this situation [19].

### Trustworthiness

The first author (IM) recruited all the informants. She has vast experience of counselling patients with pelvic pain during and after pregnancy. Generally, extensive pre-understanding may be disadvantageous during data collection and analysis in qualitative research; however, the research team was aware of this situation in order to increase conformability. The major parts of the in-depth interviews were conducted by the third author (LD) who was 'naïve' in the professional context of pelvic pain during pregnancy and the FGD was a joint interview by two authors (LD and IM). Therefore, we consider that these materials most likely reflect the views, attitudes, and perceptions of midwives currently working in antenatal care.

Level of redundancy was achieved after four in-depth interviews. In order to increase the transferability, an additional data collection was conducted in another catchment area in northern Sweden. A focus group discussion study design was selected in order to stimulate communication between informants per se and to achieve reflections from the informants on the previous findings from the first four in-depth interviews. No new major findings were revealed during the FGD. The views, perceptions and attitudes of midwives in the FGD were coherent with the findings in the previous in-depth interviews.

The degree of dependability is thought to be high due to well-organized in-depth interviews and a well-prepared focus group discussion. The focus group discussion was moderated by two persons, one with prior knowledge of the context (IM) and one who was 'naïve' in this medical area (LD). This composition of moderators most probably strengthened and stimulated the discussion in the focus group.

The absolute number of informants participating in the study (N = 10) might be considered a low number of participants and therefore a weakness in the study. However, the informants had on average 25 years of experience as midwives, mainly within antenatal care. Since more than half of pregnant women develop pelvic pain during pregnancy with different degrees of symptoms, this pregnancy-related condition is a very prevalent, medical problem which inevitably has to be dealt with by midwives within ANC. Therefore, midwives working within ANC are forced to develop knowledge and understanding of the phenomenon early during their professional career.

## Conclusions

PP was considered a common, clinical problem that had most likely increased in prevalence in recent decades and could feature prominently in a woman's experience of pregnancy. The informants had developed a strategy for supporting pregnant women affected by PP. The pregnant woman's fear of not being believed concerning her symptoms, and the risk of being regarded as a malingerer were acknowledged. Mistrust between a midwife and a woman might occur when the patient's symptoms were vague and ill defined. PP was not considered as something that complicated delivery, and women experiencing it were advised to await 'the natural course of the pregnancy'.

The current study has shown that PP is acknowledged as a prevalent maternal health care problem that requires attention and substantial support by the midwives. The midwives' own knowledge of PP was mostly self-acquired which indicates a need of education within this field of maternal health. Further, we suggest a closer collaboration between health professionals such as midwives, obstetricians, physiotherapists and social workers within the maternal health care system in order to optimize the management of women with PP.

## Competing interests

The authors declare that they have no competing interests.

## Authors' contributions

IM, AW and LD created the study design. IM, AW and LD designed the interview guidelines used for the in-depth-interviews and the focus group discussions. LD and IM performed the in-depth interviews and IM and LD conducted the focus group discussions. IM coded all data with assistance from AW and LD. The manuscript was written by all authors. All authors read and approved the final manuscript.

## Pre-publication history

The pre-publication history for this paper can be accessed here:

http://www.biomedcentral.com/1471-2458/10/600/prepub
